# Factor Structure of the Chinese Version of the Parent Adult-Child Relationship Questionnaire

**DOI:** 10.3389/fpsyg.2018.00315

**Published:** 2018-03-13

**Authors:** Daoyang Wang, Dan Dong, Peixin Nie, Cuicui Wang

**Affiliations:** ^1^Department of Psychology, Anhui Normal University, Wuhu, China; ^2^Collaborative Innovation Center of Assessment Toward Basic Education Quality, Beijing Normal University, Beijing, China; ^3^State Key Laboratory of Cognitive Neuroscience and Learning, IDG/McGovern Institute for Brain Research, Beijing Normal University, Beijing, China

**Keywords:** Chinese version of the PACQ, reliability, validity, confirmatory factor analysis, adult-children

## Abstract

The Parent Adult-Child Relationship Questionnaire (PACQ) included two identical versions of the 13-item scale, which were administered to each subject, one which referred to “relationship with mother” and the other to “relationship with father.” The PACQ, originally in English, is a self-report measure of the filial relationship. The present study aimed to develop a Chinese version of the PACQ and use it to explore Chinese parent adult-child relationships. A total of 454 Chinese adult-children completed the Chinese version of the PACQ. The structure of the questionnaire was analyzed using exploratory factor analysis (EFA) and confirmatory factor analysis (CFA). We found that the Cronbach's α was 0.66–0.88 for fathers and 0.76–0.91 for mothers, which demonstrates high internal consistency reliabilities of the Chinese version of the PACQ. The Chinese version of the PACQ for father had similar constructs similar to with those of the original English version. However, a new factor for mothers, “attachment,” was derived from the original English version. The results suggested that the Chinese version of PACQ is a valid and reliable measure of relationship quality between Chinese adult-children and their parents.

## Introduction

Parent adult-child relationships are central and important for one's whole life. This unique relationship differs from other types of social associations due to its enduring quality (Lin, 2006). Parents and adult-children usually have a positive relationship with frequent contact, attachment, emotional closeness, and obligations (Bengtson et al., 2003; Ward et al., 2009). Lum and Phares (2005) propose that the construct of emotional availability plays a key role in the quality of parent-child relations. Peisah et al. (1999) reported that the responsibility and regard are also important to parent-child relations. Although the relationship between parents and their adult-children seems influential throughout life, studies did not found consistent results on parent and adult-child relationship. The reason is that different studies used different measures to evaluate relationships between parents and their adult-children, which will result in inconsistent results (Kaufman and Uhlenberg, 1998; Lang and Schütze, 2002; Lüscher, 2003; Trommsdorff, 2006). Peisah et al. (1999) developed a Parent Adult-Child Relationship Questionnaire (PACQ) that was proved to be valid and reliable. This measurement, similar to LEAP (The Lum Emotional Availability of Parents, Lum and Phares, 2005), assesses adult-children's, children's and adolescents' perceived relationship with their mothers and father separately.

The PACQ consists of 26 items: 13 items measure mother and adult-child relationships, and 13 items measure father and adult-child relationships. The questionnaire section for mother and adult-child relationship assesses two factors (responsibility and regard), and the section for father-adult relationship assesses three factors (responsibility, regard, and control). Responsibility plays an important role in family loyalty and filial maturity, and is an important determinant for parent adult-child relationships. Responsibility is considered “support banks” among family members over the course of a lifetime (Schwarz et al., 2005). Regard refers to parents and children respect each other in a fair and consistent way, and with mutual respect, which involves sincere communication and support. In Pakistan, regard seems to be the leading factor for relationship quality of adolescents with their parents (Saeed and Hanif, 2014). Control is another important factor for parent-child relationships. Control has been described as an enduring emotional tie to a caregiver, particular for the father tie to their children. Studies indicated the PACQ is a reliable measure of relationships between adult-children and their parents (Pitzer et al., 2011).

Studies found other factors including age, gender, social economic status, and subcultural background (e.g., American Caucasian and American African; urban and rural in China) of adult-children may affect the relationship between adult-children and their parents (Julian et al., 1994; Bonsang, 2007; Cherlin, 2010; Babore et al., 2017; Li and Carter, 2017). Compared to western culture, there may be also different traditional connotations in the relationship between parents and children under Chinese cultural context. For example, in the west, most elderly tend to be more independent and live their own life, while in China, people mostly prefer living with their adult-children and depend on their offspring taking care of them (Bonsang, 2007; Li and Carter, 2017). Chinese culture is greatly influenced by Confucianism and is seen as a “culture of family” (Lin, 2006). Namely, it is a collectivistic culture rather than individualistic, as is Western culture. Traditionally, the Chinese are known for their strong family bonds. The concept of “filial piety” in Confucian philosophy, which is the respect for one's parents and elders, is seen as the foundation of family bonds and considered to be the soul of traditional Chinese culture. According to this concept, Chinese parents normally feel more sense of obligation to raise and provide an education for their children, and children in turn have the duty to support their parents. In other word, parents and children in China are attached tighter to each other due to culture environment.

Previous studies which focused on the parent-child relationship have some limitations (Kaufman and Uhlenberg, 1998; Ikkink et al., 1999; Lang and Schütze, 2002; Lüscher, 2003; Trommsdorff, 2006). Previous studies focused only on young children and few studies have examined the relationships between parents and their adult-children. While one study did focus on this parent adult-child relationship, the sample was from metropolitan areas of Mainland China, such as Beijing, and rural areas have been overlooked (Lin, 2006). The distinction between rural participants and urban participants is based on *hukou* (the official Chinese household registration, in which rural participants have the agricultural *hukou* while urban participants have the non-agricultural *hukou)* (Wang et al., 2017). In China, urban and rural areas are different in many conditions (e.g., economy), which may affect adult-children's evaluation of parent-child relationship. Thus, the present study aimed to explore the relationship between parents and their adult-children in Chinese urban and rural areas. In addition, there is only an English version of the PACQ. Multiple language versions of the PACQ, such as in Mandarin Chinese, will allow a larger and more varied population who have limited English proficiency to be studied. Thus, the present study developed a Chinese version of the PACQ and used it to explore Chinese parent adult-child relationships.

The main aim of the present study was to explore the factor structure of the Chinese version PACQ. The more specific aims were as followed: (a) to examine whether the same factor structure fits across the Chinese version and the original English version; (b) to investigate the reliability of the Chinese PACQ.

## Materials and methods

### Sample and data collection

The survey was conducted between March and April in 2016. A total of 454 adult-children participants (72% are females) from Anhui province participated in the study. Anhui is a province in middle-east of China, whose social and economic development is in an average level. The adult-children participants include: college students (one university) and the parents of primary school students (one primary school). One class was selected randomly at each grade level. Data collection was conducted in computer room of the collaborative school, via an online questionnaire tool, by the research group (two doctorate tutors and three master's graduate students). All participants signed an informed consent form and were paid for their participation. The study was reviewed and approved by the Institutional Review Board of Human Research Ethics Committee for Non-Clinical Faculties of CICA-BEQ at Beijing Normal University.

The descriptive statistics of participants were showed in Table 1. We randomly divided 454 participants into two subgroups (see section Statistical Analysis for details). There was no significant difference between the two subgroups in age, education level, gender and *hukou* (*p* > 0.05).

**Table 1:** Descriptive statistics of participants' background variables.

**Participants**	**Total (*N* = 454)**	**Gender**		**Randomly grouped**		***t***
		**Female (*n* = 327)**	**Male (*n* = 127)**	**Group 1 (*n* = 223)**	**Group 2 (*n* = 23)**	
**AGE**						
*M*±*SD* range	22.98 ± 7.04 (18–48)	22.04 ± 5.66 (18–48)	25.47 ± 9.40 (18–48)	23.07 ± 6.93 (18–48)	22.89 ±7.16 (18–48)	0.28
**YEARS OF EDUCATION**						
*M*±*SD* range	14.65 ± 1.87 (5–19)	14.84 ± 1.44 (5–19)	14.16 ± 2.61 (5–19)	14.75 ± 1.70 (5–19)	14.16 ± 2.61 (5–19)	1.16
*hukou*						*χ^2^*
Non-agricultural	30.4%	29.1%	33.9%	29.1%	31.6%	0.57
Agricultural	69.6%	70.9%	66.1%	70.9%	68.4%	
**GENDER**						
Female	72.0%	—	—	72.2%	71.9%	0.94
Male	28.0%	—	—	27.8%	28.1%	

*Gender, male = 0, female = 1; hukou, non-agricultural = 0, agricultural = 1*.

### Instruments

The PACQ included two identical versions of the 13-item scale: one is “the relationship with mother” and the other is “the relationship with father” (Table 2; Peisah et al., 1999). The original English version of the PACQ includes the dimensions of regard (five items) and responsibility (eight items) for the mother, and the dimensions of control (five items), regard (four items), and responsibility (four items) for the father. Each item is a statement that described the relationship with the mother or father (e.g., “I look forward to seeing my mother/father”; “I feel responsible for my mother's/father's happiness”), and participants were asked to indicate the extent to each statement generally described themselves using a 4-point scale, with anchors of 0 (not true at all) and 3 (very true). The English version of the PACQ is a reliable self-report measure of the filial relationship, as shown by Peisah et al. (1999); the study reported for the mother's section, Cronbach's coefficient was 0.87 for regard and 0.82 for responsibility, and for the father's section, Cronbach's coefficient was 0.86 for regard, 0.74 for responsibility, and 0.87 for control.

**Table 2:** PACQ for parent adult-children relationship.

**Adult-children participants**	**PACQ for father**	**PACQ for mother**
Adult man (son)	Father-son relationship	Mother-son relationship
Adult woman (daughter)	Father-daughter relationship	Mother-daughter relationship

The PACQ was translated into Chinese with a Chinese graduate student who majored in English, and fidelity was ensured through back translation with a native English speakers. Discrepancies were discussed until an agreement was reached between the authors, English major graduate student and native English speaker. This version was then refined, using well-known words and easy grammar to ensure that questionnaire items could be easily understood by respondents who come from development education level.

### Statistical analysis

Responses to all items were subjected to principal component factor analysis. SPSS 22.0 (Chicago, IL, USA) and MPLUS 7 (Los Angeles, CA, USA) were used for analysis.

Data analysis included the calculation of Cronbach's α for each item and the identification of psychometrically weak items. Partial eta squared was used as an estimate of effect size when interpreting multivariate analysis of variance (MANOVA) results. A principal factor analysis and Pearson correlation coefficients between subscale scores were also conducted. Cross-validation analyses were carried out since participants were divided randomly into two subgroups (group 1 and group 2). Exploratory factor analysis (EFA) was performed for group 1, and confirmatory factor analysis (CFA) was performed for group 2. In the group 1, to identify the number of reserved components, Cattell's scree test (Cattell, 1966) and parallel analysis (PA, Horn, 1965) were performed for the PACQ responses of fathers and mothers separately. In the group 2, we examined the factor structure of the Chinese version of PACQ by performing CFA.

The following indices were used to assess the model fit: χ^2^/df, the root mean square error of approximation (RMSEA), comparative fit index (CFI), and Tucker-Lewis index (TLI). According to generally accepted criteria, RMSEA values less than 0.05 would be considered good, between 0.05 and 0.08 would be considered adequate, and between 0.08 and 0.10 would be considered mediocre (Schermelleh-Engel et al., 2003). A good fit would be indicated by a CFI more than 0.95 and a TLI more than 0.95, and an acceptable fit would be indicated by a CFI more than 0.90 and a TLI more than 0.90 (McDonald and Ho, 2002). Internal consistencies were assessed by Cronbach's α coefficient. The statistical analysis employs χ^2^ differences (Δχ^2^) to compare models, with non-significant values indicating that the new model is better. Because the χ^2^ difference test is highly dependent on the number of subjects (Schweizer, 2010), two other indices of CFI differences (ΔCFI) and TLI differences (ΔTLI) were used. The cut-off criteria for ΔCFI and ΔTLI is usually 0.01; ΔCFI or ΔTLI greater than 0.02 indicates definite differences (Meade et al., 2008). The raw data and statistical syntax can be obtained from the author.

## Results

### Exploratory factor analysis of the PACQ

**Figure 1:**
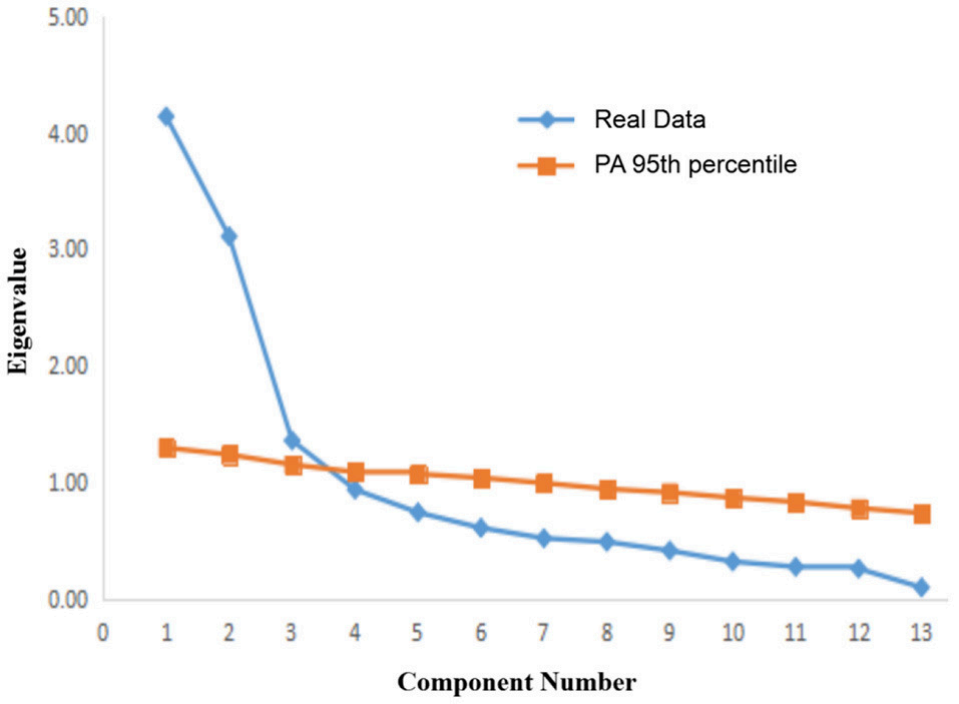
Plot of the first 13 eigenvalues on PACQ for father.

Based on the EFA results of the group 1, separate principal component analyses were performed for the father and mother sections of the Chinese version. Regarding the Chinese version of the PACQ for fathers, Horn (1965) recommended the Cattell's scree test with a PA to retain only those factors whose eigenvalues are greater than those of the random data (see Figure 1). There was a clear discontinuity in the eigenvalues between the third and the fourth factors in the scree plot, and only the first third whose eigenvalues are greater than the random data. Therefore, we chose the three-factor solution. The three-factor solution accounted for 67.90% of the variance in the PACQ for fathers. These three factors consisted of control, regard, and responsibility. The control factor accounted for 31.44% of the variance (eigenvalue: 4.09), the regard factor accounted for 27.38% of the variance (eigenvalue: 3.56), and the responsibility factor accounted for 9.07% of the variance (eigenvalue: 1.18). These results are similar to those of the original English version (Peisah et al., 1999).

As shown in Table 3, most of the item scores had loadings on their expected theoretical parent adult-child relationship for fathers, with loading values >0.50, except for item 6 (responsibility). Item 6 positively loaded on responsibility with a loading value of 0.47, and on regard with a loading value of 0.77. The factor loadings for the items contributed to these factors for sons and daughters combined, as well as the factor loadings derived separately for sons and daughters (Table 3). The results of item loadings were similar for sons and daughters. The loading value of item 6 was <0.50 for the paternal relationships with sons and daughters, and positively loaded on responsibility for paternal relationships with sons and daughters, with a loading value of 0.49 and 0.38, and on regard with a loading value of 0.62 and 0.68.

**Table 3:** Principal Factor analysis of the PACQ for father.

**Group 1 (*n* = 223)**	**Factor loading**								
	**All**			**Sons**			**Daughters**		
**FACTOR 1: CONTROL**									
1. If I don't do things my father's way he will nag me.	**0.91**	−0.01	0.33	**0.94**	0.07	0.33	**0.85**	−0.10	0.31
4. I feel that my father tries to manipulate me.	**0.91**	−0.01	0.33	**0.94**	0.07	0.33	**0.85**	−0.10	0.31
7. My father tries to dominate me.	**0.79**	−0.01	0.17	**0.86**	0.1	0.27	**0.84**	−0.01	−0.02
8. I feel that my father makes too many demands on me.	**0.77**	0.04	0.02	**0.79**	−0.07	0.20	**0.85**	0.10	−0.22
11. I don't discuss much with my father because I' m afraid of being criticized.	**0.70**	0.01	0.04	**0.51**	−0.39	−0.13	**0.71**	0.12	−0.13
Eigenvalues/Explained variance (%)	4.09/31.44%			4.29/32.98%			4.23/32.52%		
**FACTOR 2: REGARD**									
2. I respect my father's opinion.	−0.09	**0.85**	0.04	−0.05	**0.89**	0.29	−0.09	**0.79**	−0.07
5. I look forward to seeing my father.	−0.03	**0.89**	0.05	−0.16	**0.88**	0.20	0.04	**0.84**	−0.01
9. I know I can rely on my father to help me if I need him.	0.12	**0.80**	0.09	0.17	**0.64**	0.42	0.18	**0.67**	−0.01
12. I don't mind putting myself out for my father.	0.01	**0.85**	0.14	−0.01	**0.89**	0.41	0.07	**0.85**	0.02
Eigenvalues/Explained variance (%)	3.56/27.38%			3.53/27.15%			3.17/24.34%		
**FACTOR 3: RESPONSIBILITY**									
3. Something will happen to my father if I don't take care of him.	0.16	0.10	**0.85**	0.25	0.29	**0.82**	0.32	−0.01	**0.69**
6. I feel responsible for my father's happiness.	−0.04	**0.77**	0.47	0.10	**0.62**	0.49	−0.01	**0.68**	0.38
10. If I don't see my father for a week I feel guilty.	0.09	0.35	**0.72**	0.14	0.35	**0.89**	0.24	0.41	**0.68**
13. My father thinks I' m good in a crisis so he calls on me all the time.	0.19	0.08	**0.64**	0.31	0.32	**0.72**	0.29	0.14	**0.78**
Eigenvalues/Explained variance (%)	1.18/9.07%			1.22/9.38%			1.12/8.60%		

*Value ≥ 0.50 are shown in bold*.

**Figure 2:**
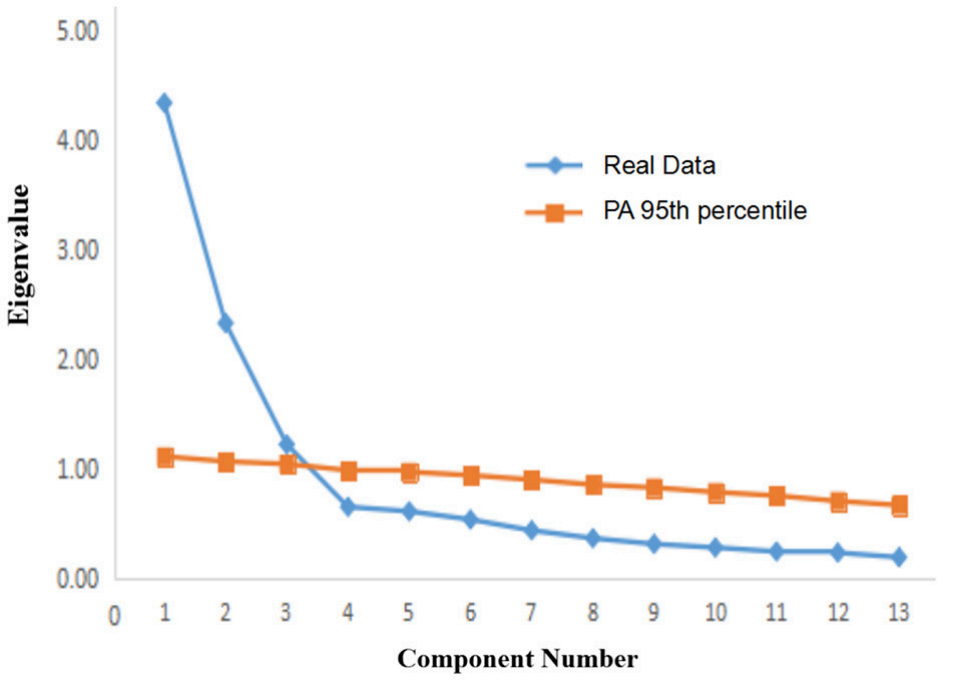
Plot of the first 13 eigenvalues on PACQ for mother.

Regarding the Chinese version of the PACQ for mothers, a plot of the first 13 eigenvalues is presented in Figure 2. There was a clear discontinuity in the eigenvalues between the third and the fourth factors. The Cattell's scree test with PA indicated a three-factor solution, which accounted for 58.04% of the variance in the PACQ results for mothers. These three factors were regard, responsibility, and attachment. The results for the regard and responsibility factors are similar to those of the original English version of the PACQ for mothers (Peisah et al., 1999). However, the responsibility factor was separated into two factors, namely, responsibility (items 2, 3, and 10) and anonymous factor (items 5, 7, 8, 11, and 13). From the perspective of Chinese culture, the meaning of the items (e.g., “my mother relies on me too much”) in the anonymous factor was more about the emotional dependence of the mother on the adult child, which is very close to the attachment (Kerns and Brumariu, 2014). So, this additional factor termed “attachment.” The regard factor accounted for 34.09% of the variance (eigenvalue: 4.43), the responsibility factor accounted for 16.11% of the variance (eigenvalue: 2.09), and the attachment factor accounted for 7.84% of the variance (eigenvalue: 1.08). As shown in Table 3, most of the item scores had loadings on their expected theoretical parent adult-child relationship for mothers, with loading values of >0.50. The factor loadings for the items contributing to these factors for sons and daughters were combined, as well as the factor loadings derived separately for sons and daughters (Table 4). The results showed that item loadings were similar for sons and daughters. The loading value of all items were >0.50 for the paternal relationships with daughters, while the loading value of item 5 and item 7 (attachment) were <0.50 for the paternal relationships with sons. The item 5 not only loaded on attachment with a loading value 0.48, but also on regard with a loading value −0.49. And item 7 not only loaded on attachment with a loading value 0.49, but also on responsibility with a loading value 0.37.

**Table 4:** Principal Factor analysis of the PACQ for mother.

**Group 1 (*n* = 223)**	**Factor loading**								
	**All**			**Sons**			**Daughters**		
**FACTOR 1: REGARD**									
1. I look forward to seeing my mother.	**0.76**	0.23	0.01	**0.82**	−0.02	−0.03	**0.72**	0.13	−0.04
4. My mother is my best friend.	**0.79**	0.14	0.21	**0.86**	0.01	0.07	**0.75**	−0.08	0.19
6. My mother shows her appreciation of me.	**0.73**	0.15	0.20	**0.82**	0.18	0.03	**0.72**	−0.12	0.16
9. I respect my mother's opinion.	**0.89**	0.17	−0.01	**0.90**	−0.09	0.03	**0.88**	−0.03	−0.12
12. I am glad to be able to repay my mother for all the love and care she gave me as a child.	**0.80**	0.27	0.02	**0.88**	0.03	0.03	**0.76**	0.16	−0.08
Eigenvalues/Explained variance (%)	4.43/34.09%			4.97/38.24%			4.32/33.23%		
**FACTOR 2: RESPONSIBILITY**									
2. I feel responsible for my mother's happiness.	0.24	**0.81**	0.14	−0.02	**0.80**	−0.02	0.02	**0.79**	0.11
3. I feel that I should take care of my mother because she has suffered so much in her life.	0.22	**0.85**	0.01	−0.03	**0.89**	−0.05	−0.06	**0.83**	−0.06
10. I feel that I have to protect my mother.	0.41	**0.68**	0.09	0.39	**0.76**	−0.03	0.33	**0.65**	0.04
Eigenvalues/Explained variance (%)	2.09/16.11%			2.40/18.48%			1.99/15.32%		
**FACTOR 3: ATTACHMENT**									
5. My mother's difficulty in making decisions has been a burden on me.	−0.33	0.08	**0.58**	−0.49	0.19	0.48	−0.40	0.05	**0.63**
7. I am the only one my mother can rely on.	0.06	0.11	**0.57**	−0.05	0.37	0.49	0.02	0.01	**0.61**
8. My mother thinks I am good in a crisis so she calls on me all the time.	0.16	0.13	**0.58**	0.11	0.16	**0.66**	0.10	0.08	**0.54**
11. My mother relies on me too much.	0.14	0.02	**0.81**	0.08	−0.15	**0.94**	0.11	0.04	**0.77**
13. I feel like I parent my mother.	0.14	−0.07	**0.72**	0.01	−0.18	**0.78**	0.19	−0.10	**0.72**
Eigenvalues/Explained variance (%)	1.08/7.84%			1.20/9.21%			1.00/7.44%		

*Value ≥ 0.50 are shown in bold*.

### Confirmatory factor analysis of the PACQ

Based on the CFA results of the group 2, separate CFA were performed for the father and mother sections of the Chinese version. Regarding the Chinese version of the PACQ for fathers, we first tested the original three-factor model (Model I). Because item 6 was positively loaded on responsibility with a loading value of 0.47, and on regard with a loading value of 0.77 and a loading value >0.50, item 6 was re-categorized from a responsibility factor to a regard factor. Then, we tested the revised three-factor model (Model II). Furthermore, the modification model indices for the three-factor model indicated that responsibility and regard factor may have been cross-loadings for item 6. Model misfit could be a result of failing to specify item cross-loadings (Marsh et al., 2010). Therefore, we freed freeing paths the responsibility item 6 and the regard factor (Model III). Regarding the results of the Chinese version of the PACQ for mothers, we first tested the original two-factor (regard and responsibility) model (Model IV). Second, we tested the revised three-factor (regard, responsibility, and attachment) model (Model V). Finally, we tested a hierarchical model assuming the unidimensionality of the measure (i.e., PACQ for father original three-factor and PACQ for mother original two-factor) (Model VI).

The results of the PACQ for father indicated that the Model II fit the data considerably better than the Model I (ΔCFI = 0.038 and ΔTLI = 0.047; Table 3). Although the fitting index in Model III was higher than the Model II, it was not significant (ΔCFI = 0.006 and ΔTLI = 0.008). Therefore, the Model II was more suitable for analyzing father and adult-child relationship in a Chinese cultural background. The results of the PACQ for mother indicated that the Model V fit the data significantly better than the Model IV [Δχ^2^_(2)_ = 666.85, *p* < 0.001; ΔCFI = 0.074 and ΔTLI = 0.087; Table 5]. Meanwhile, the three-factor model with double loading fit the data considerably better than the three-factor model [Δχ^2^_(2)_ = 357.28, *p* < 0.001; ΔCFI = 0.051 and ΔTLI = 0.049]. Thus, the three-factor model with double loading was more suitable for analyzing mother and adult-child relationship results in a Chinese cultural background.

**Table 5:** Comparison of several PACQ fit indices for the estimated models.

**CFA models**	***χ^2^***	***df***	***χ^2^*/*df***	***Δχ*^2^**	**Δ*df***	**CFI**	**TLI**	**ΔCFI**	**ΔTLI**	**RMSEA**
**PACQ FOR FATHER**										
Model I: Original three-factor model	595.90	62	9.61	—	—	0.915	0.893	—	—	0.138
Model II: Revised three-factor model	358.32	62	5.78	—	—	0.953	0.940	0.038	0.047	0.103
Model III: Revised three-factor model with double loading	315.29	61	5.17	43.03^***^	1	0.959	0.948	0.006	0.008	0.096
**PACQ FOR MOTHER**										
Model IV: Original two-factor model	1264.01	64	19.75	—	—	0.867	0.838	—	—	0.203
Model V: Three-factor model	597.16	62	9.63	666.85^***^	2	0.941	0.925	0.074	0.087	0.138
**PACQ FOR FATHER AND MOTHER**										
Model VI:hierarchical model	6590.18	299	22.04	—	—	0.553	0.514	—	—	0.215

n = 231 (group 2); CFA, confirmatory factor analysis; CFI, comparative fit index; TLI, Tucker-Lewis index; RMSEA, root mean square error of approximation; χ^2^/df, the associated p-values were always < 0.001.

Model I: PACQ for father original three-factor (control, regard, and responsibility) model.

Model II: PACQ for father original three-factor model, and separate item 6 from responsibility factor to regard factor.

Model III: PACQ for father original three-factor model, and freeing paths between the responsibility item 6 and the regard factor.

Model IV: PACQ for mother original two-factor (regard and responsibility) model.

Model V: PACQ for mother original three-factor (regard, responsibility, and attachment) model.

Model V: a hierarchical model assuming the unidimensionality of the measure (i.e., PACQ for father original three-factor and PACQ for mother original two-factor).

*** *p < 0.001*.

The loadings of each item on the corresponding latent construct of the PACQ for mothers and fathers of the three-factor model are reported in Tables 6, 7. All loadings for the items on the corresponding latent variables were statistically significant (all, *p* < 0.01). Only one item with a potentially low loading (<0.50) was identified, which was from the PACQ for mothers of the attachment dimension item 5 (*r* = 0.40). Although factor loading of this item was below 0.50, the signs of the loadings were in the correct direction. Moreover, the regard, responsibility, and attachment/control variables were correlated with each other (range, *r* = 0.11–0.66). The most significant positive correlation was between responsibility and regard (*r* = 0.66, *SE* = 0.036).

**Table 6:** Confirmatory factor analysis and factor correlations based on responses to the PACQ for father.

**Item No**.					**CFA Standardized loading**		
					**Control**	**Regard**	**Responsibility**
1. If I don't do things my father's way he will nag me.					0.86^**^		
4. I feel that my father tries to manipulate me.					0.86^**^		
7. My father tries to dominate me.					0.86^**^		
8. I feel that my father makes too many demands on me.					0.81^**^		
11. I don't discuss much with my father because I' m afraid of being criticized.					0.67^**^		
2. I respect my father's opinion.						0.85^**^	
5. I look forward to seeing my father.						0.90^**^	
6. I feel responsible for my father's happiness.						0.83^**^	
9. I know I can rely on my father to help me if I need him.						0.70^**^	
12. I don't mind putting myself out for my father.						0.88^**^	
3. Something will happen to my father if I don't take care of him.							0.53^**^
10. If I don't see my father for a week I feel guilty.							0.81^**^
13. My father thinks I' m good in a crisis so he calls on me all the time.							0.66^**^
					**Correlation between latent variables**		
**Dimensions**	**No. of items**	**Mean**	***SD***	α	**Control**	**Regard**	**Responsibility**
Control	5	4.78	3.41	0.88	1.00		
Regard	5	10.74	3.52	0.88	−0.11^*^	1.00	
Responsibility	3	3.51	1.96	0.68	0.45^**^	0.56^**^	1.00

*Revised three-factor model, Model fit results: χ^2^ = 358.32, df = 62, comparative fit index (CFI) = 0.953; Tucker–Lewis Index (TLI) = 0.940; root mean square error of approximation (RMSEA) = 0.103*.

*PACQ for father: Control scale (item 1, 4, 7, 8, 11); Regard Scale (item 2, 5, 6, 9, 12); Responsibility Scale (item 3, 10, 13)*.

* p < 0.05,

** *p < 0.01*.

**Table 7:** Confirmatory factor analysis and factor correlations based on responses to the PACQ for mother.

**Item No**.					**CFA Standardized loading**		
					**Regard**	**Responsibility**	**Attachment**
1. I look forward to seeing my mother.					0.82^**^		
4. My mother is my best friend.					0.81^**^		
6. My mother shows her appreciation of me.					0.78^**^		
9. I respect my mother's opinion.					0.88^**^		
12. I am glad to be able to repay my mother for all the love and care she gave me as a child.					0.84^**^		
2. I feel responsible for my mother's happiness.						0.85^**^	
3. I feel that I should take care of my mother because she has suffered so much in her life.						0.80^**^	
10. I feel that I have to protect my mother.						0.72^**^	
5. My mother's difficulty in making decisions has been a burden on me.							0.40^**^
7. I am the only one my mother can rely on.							0.57^**^
8. My mother thinks I am good in a crisis so she calls on me all the time.							0.68^**^
11. My mother relies on me too much.							0.80^**^
13. I feel like I parent my mother.							0.64^**^
					**Correlation between latent variables**		
**Dimensions**	**No. of items**	**Mean**	***SD***	α	**Regard**	**Responsibility**	**Attachment**
Regard	5	10.86	3.61	0.91	1.00		
Responsibility	3	7.02	2.08	0.83	0.66^**^	1.00	
Attachment	5	5.82	3.10	0.76	0.34^**^	0.29^**^	1.00

Three-factor model, Model fit results: χ^2^ = 597.16, df = 62, comparative fit index (CFI) = 0.941; Tucker–Lewis Index (TLI) = 0.925; root mean square error of approximation (RMSEA) = 0.138

PACQ for mother: Regard Scale (item 1, 4, 6, 9, 12); Responsibility Scale (item 2, 3, 10); attachment scale (item 5, 7, 8, 11, 13).

^*^p < 0.05,

** *p < 0.01*.

### Internal consistency

Descriptive data for the Chinese version of the PACQ for fathers had Cronbach's α coefficients of 0.88 for control, 0.88 for regard, and 0.68 for responsibility, and the PACQ for mothers had Cronbach's α coefficients of 0.76 for attachment, 0.91 for regard, and 0.83 for responsibility (see Tables 5, 6). The scales of the Chinese version thus maintained an internal consistency that was similar to that of the original English version of the PACQ (Peisah et al., 1999).

### The effects of age, gender, and education level on the PACQ results

We explored the effects of adult-child characteristics (age, gender, education level, *hukou*) on the relationship between adult-child and their parents. Dependent variables included three dimensions of the PACQ. The results showed that adult-child characteristics were likely to affect the relationship between adult-child and their parents. The results were based on Pillai's trace. Looking at results for father and mother respectively, there were no significant main effects for fathers. For mothers, main effect was only significant in *hukou* (*p* < 0.05). Based on the univariate ANOVA, *hukou* also had a significant impact on responsibility [*F*_(1, 223)_ = 3.18, *p* < 0.05, partial η^2^ = 0.033]. By looking at the mean values, an agricultural *hukou* was significantly associated with higher scores (agricultural vs. non-agricultural, (7.28 ± 1.90) vs. (6.45 ± 2.34), *t* = 3.96, Cohen' *d* = 0.40).

## Discussion

The present study developed the Chinese version of the PACQ for the first time and assessed its reliability and construct validity. We found that the Chinese version of the PACQ is reliable and suitable for the assessment of Chinese parents and their adult-children relationship. The original PACQ and the Chinese version of the PACQ both showed favorable psychometric properties in terms of reliability (Cronbach's α was 0.74~0.87; Peisah et al., 1999, p. 32). In the present study, the Cronbach's α coefficient of Chinese version PACQ for each dimension was acceptable (0.68~0.91). In addition, the dimensions of the Chinese version of the PACQ for fathers were regard, responsibility and control, which were similar to the original English version. However, for mothers, a new factor “attachment” was derived from the original English version.

The Chinese version of PACQ assesses three factors for father (responsibility, regard, and control), and it's also assesses three factors for mother (responsibility, regard, and attachment). Both Chinese father and mother are responsible for their children and with mutual respect. However, most Chinese fathers often take much more time on the work than family, while is very different with the father in Western culture who pay more attention on the interaction with children. A survey found that most Chinese fathers have little time with children, and that mothers tend to play as a leading factor in children's development (Wu et al., 2017). This may lead to the relatively more attachment between children and mothers, than those with fathers (Bureau et al., 2017). Consequently the emerging factor “attachment,” which was derived from the original English version for mother, may be result from these factors.

Atkinson (1989) proposed the attachment theory that mother represents security and the typical source of a child's initial attachment and identification. Researchers found that the attachment was related with children's behavior and mental health (Bovenschen et al., 2016; Pallini et al., 2017). Insecure attachment between mother and children may be a risk factor in children's development (Cicchetti and Greenberg, 1991). Mother is the first significant attachment figure in a person's life, and this attachment persists throughout the whole life. In Chinese culture, mother plays an important role in the family values and parent adult-child relationship (Ainsworth, 1969; Chaplin et al., 2005; Schwarz et al., 2005; Macfie et al., 2014). Chinese children depend more on their mothers in their study in school and daily life. Thus, in addition to responsibility and regard, attachment is also important for the relationship between mother and their adult-child in Chinese culture.

Regarding the PACQ for fathers, item 6 (“I feel responsible for my father's happiness”) was considered under “responsibility” in the English version, but it was considered under “responsibility” and “regard” in the Chinese version. Responsibility in this context refers to guilt, burden, and protectiveness, whereas regard refers to attachment and care. This result suggests that, as well as concerning about their fathers' happiness consistent with that in Western culture, Chinese adult-children also feel the duty of providing with more care for their fathers. Huff (2015) found that “for my father's happiness” may be particularly linked to REN (仁, meaning Mercy, Gentle, Kindness, loving, Caring) that is highlighted by Mencius theory (Man is naturally good) as a priori for Chinese people's personal accomplishment.

The study firstly developed the Chinese version of the PACQ and explored the parent adult-child relationship in urban and rural areas in China. We found agricultural *hukou* was significantly associated with mother's responsibility. This may result from the fact that China's rural insurance system for older people started late and was still incomplete (Holroyd, 2003; Rokicki and Donato, 2016). Thus, rural adult-children are more responsible for raising parents. Compared with the original English version, the Chinese version of the PACQ showed better validity and similar reliability coefficients, which indicated the Chinese version of the PACQ is suitable for assessing Chinese parent adult-child relationships. In addition, a new factor “attachment” was derived from the original English version, which indicated the relationship between mother and their adult-child were close with each other.

The study has some limitations. Firstly, the present sample was from Anhui province only, future studies should therefore recruit participants from other provinces in China. Secondly, we did not test convergent validity in this study, and future studies should to verify the external validity of the Chinese version of the PACQ. Finally, we recommend that future versions of the PACQ consider testing different items for the scales to improve the variety, specifically, measurement invariance across Chinese and Western cultures in adult samples.

## Author contributions

DW, Guarantor of integrity of entire study, Study concepts, Study design, Literature research, Data acquisition, Data analysis/interpretation, Statistical analysis, Manuscript preparation, Manuscript final version approval. DD, Literature research, Data acquisition, Manuscript preparation, Manuscript final version approval. PN, Literature research, Manuscript editing, Manuscript revision, Manuscript final version approval. CW, Literature research, Guarantor of integrity of entire study, Study design, Manuscript definition of intellectual content, Manuscript editing, Manuscript revision, Manuscript final version approval.

### Conflict of interest statement

The authors declare that the research was conducted in the absence of any commercial or financial relationships that could be construed as a potential conflict of interest.
